# 

**Published:** 1844-06

**Authors:** 


### WESTERN JOURNAL

OF

### MEDICINE AND SURGERY.

### EDITORS.

---

DANIEL DRAKE, M. D.,

PROFESSOR OF PATHOLOGY AND THE PRACTICE OF MEDICINE IN THE MEDICAL INSTITUTE OF LOUISVILLE:

LUNSFORD P. YANDELL, M. D.,

PROFESSOR OF CHEMISTRY AND PHARMACY IN THE SAME:

THOMAS W. COLESCOTT, M. D.

### INDEX TO VOL. I.

[NEW SERIES.]

A

Abdominal tumor, 527.
Abscess, hepatic, case of, 377.
Abscess with loss of a kidney, case of, 119.
Air, purification of, 178.
American origin of syphilis, 360.
Anatomy of hernia, 448.
Anderson's case of difficult labour, 121.
Animalcules in the intestinal canal, 533.
Aphonia, complete, 19.
Aran on disease of the heart, 46.
Atlas, anatomical, 91, 375.
Automaton, articulated, 359.
Anchylosis of the knee joint, 502.
Anus, open, 521.

B

Bache's introductory lecture, 90.
Belladonna, employment of, 75.
Billing's first principles of medicine, 421.
Black tongue, 508.
Boling's case of cataract, 17.
Bone maxillary, extirpation of, 223.
Boston, mortality of, 179.
Burning wells of Kenawha, 373.
Burr's operation for anchylosis, 502.
Brodie, Sir B., on fistula in ano, 535.

C

Cancerous diseases, increasing frequency of, 356.
Cancer of the lip and jaw, 405.
Castration, performed on the patient by himself, 179.
Catamenia, influence of opium upon, 164.
Cataract, with tremulous iris, 17.
Chailly's midwifery, 513.
Chicago, medical school of, 185.
Chlorosis, 534.
Cyclopœdia of practical medicine, 514.
Clinkers, use of, 97.
Cod liver oil, capsules of, 359.
Consumption, cure of, 21.
Cooper on dislocation of the joints, 72.
Cooper on hernia, 448.
Cough, remedy for, 76.
Cure of consumption, McDowell on, 21.
Cynosis, pathological causes of, 267.
Consumption, naphtha in, 523.

D

Del Valle on extirpation of the maxillary bone, 223.
Diabetes mellitus, treated by iodurет of iron, 273.
Dipsopathia, the latest medical humbug, 176.
Disease, magneto-electricity in, 279.
Diseases of Tennessee, 105.
Dissector, Wilson's, 92.
Drake's speculations on mesmerism, 285.
Dunglison's human physiology, 450.
Dupierris' operation for cancer, 405.

Dupierris on complete aphonia, 19.
Dyspepsia, Yandell's case of, 219.
Dowler on post-mortem fever, 470.

E

Elegant sticking plaster, 76.
Electricity in poisoning, 517.
Elliotson's principles and practice of medicine, 340.
Epidemic scarlatina, belladonna in, 75.
Epilepsy, case of, 384.
Euphorbia corollata, 86.
Equivocal generation, 514.
Erysipelas, 508.
Experiments on p. m. fever, 470.

F

Face-ache, treatment of, 277.
Facial neuralgia, veratria in, 358.
Fair at a lunatic asylum, 178.
Females co-twins with males, infecundity of, 277.
Ferruginous preparation, use of, 97.
Fever, nervous, 386.
Filing, use and abuse of, 77.
Food and diet, Pereira on, 123, 440.
French gratitude to medical men, 79.
Frequency of hernia, 315.
Fever, post-mortem, 470.
Fistula in ano, 535.

G

Ganglionic typhus, 386.
Gas, noisy expulsion of, from the vagina, 78.
Generation, equivocal, 514.
Goddard on the human teeth, 410.
Goodwin's case of tubercular disease, 382.
Gravity *vs.* hilarity, 175.
Great vessels, diseases of, 46.
Gregory on small pox, 166.

H

Hall, Prof., of Baltimore, 556.
Heart, diseases of, 46.
Hemorrhage, proximate causes of, 352.
Hepatic abscess, case of, 377.
Hernia, anatomy of, 448.
Hooping Cough, treatment of, 180.
Hospitals of Philadelphia, 459.
Human Physiology, 450.
Human teeth, anatomy of, 410.
Hydrocephalus, cure of, by hydriodate of potassa, 269.
Hysteria, tobacco in, 272.
Hydriodate of potassa, 269.

I

Indiana, lunatic asylum of, 466.
Inferior lip and jaw, cancer of, 405.
Infant subject, opium upon, 157.
Introductory of Bache, 90.
Introductory of Litton, 183.
Insanity, Jarvis on, 181.
Inversion of uterus, remarks on, 1.
Ioduret of iron, used in diabetes mellitus, 273.
Iron, ioduret of, in diabetes, 273.
Italian medicine, remarks on, 363.

J

Jarvis on insanity of free blacks, 181.
Joints, dislocation of, 72.
Journal, Western, 82, 188.

K

Knee joint, anchylosis of, 502.
Kenawha, burning wells of, 373.
Kentucky, lunatic asylum of, 87.
Kilpatrick's case of hepatic abscess, 377.
Kilpatrick's case of anchylosis, 502.

L

Life preserver, 186.
Litton, introductory of, 183.
Louisiana, medical college of, 184.
Louisville, medical institute of, 187, 280.
Lunatic hospitals, 369.
Lunatic asylum of Kentucky, 87.
Lunatic asylum of Indiana, 466.
Lunatic asylum of New York, fair at, 178.
Lungs, scrofulous affections of, 353.

M

Magendie's physiology, 283.
Malgaigne on the frequency of hernia, 315.
Magneto-Electricity in various diseases, 274.
Materia medica, Pereira's treatise on, 24.
McDowell's cure of consumption, 21
Mobile, topography of, 546.

McMurtry on a new ferruginous preparation, 97.
Medical practitioners, judicious advice to, 453.
Medical classes, 187.
" men, French gratitude to, 79.
Medical men, poverty of, 174.
" ethics, 184.
" school, catalogues of, 283.
" " of Transylvania, 460.
Medical school of Chicago, 185.
" " of Nashville, 183.
" department of Saint Louis University, 282.
Medical institute of Louisville, 280.
Medical Journal of Illinois, 554.
Medicine, principles and practice of, 340.
Medicine, first principles of, 421.
Medicine, principles of, 144.
Medicines, advantage of, in liquid form, 358.
Medicines administered by nose, 457.
Memory of Sir C. Bell, compliment to, 359.
Mesmerism, 187, 368.
Mesmeric Somniloquism, report on, 285.
Mercury in scrofulous affections of the lungs, 353.
Midwifery, statistics of, 454.
Milk injected into the veins, 177.

N

Nervous fever, 386.
New medical journal, 183.
New York, lunatic asylum of, 178.
Noeggerath's observations, 431.
Nose, medicines administered by, 457

O

Œdopsophia, 78.
Opium, effects of, upon infants, 157.
Opium, influence of, upon catamenia, 184.
Origin of syphilis, 360.

P

Paris' pharmacologia, 281.
Patterson's valedictory address, 463.
Pereira's materia medica, 24.
Pereira's treatise on food and diet, 123, 440.
Pessaries, objections to, 277.
Pharmacologia, Paris', 281.
Philadelphia, hospitals of, 459.
Phthisis and intermittent fevers, antagonism of, 176.
Phthisis and tubercles, 455.
Physiology of Magendie, 283.
Physic, practice and principles of 228.
Pregnancy, signs of, 278.
Preserving health, natural means of, 83.
Principles and practice of physic, 228.
Professor Hall, impeachment of, 284.
Professional success, 175.
Protracted and difficult labor, case of, 121.
Prout on stomach and renal diseases, 250.

Q

Quinine, effects of large doses, 362.

R

Renal calculi, case of, 119,
Renal diseases, treatment of, 250.
Report on mesmeric somniloquism, 285.
Richardson on the diseases of Tennessee, 105.

S

Schoenlein on typhus, 386.
Scrofulous affections, mercury in, 353.
Secretory organs, sulphur water on, 276.
Sheffield, vital statistics of, 171.
Sibson on thoracic and abdominal viscera, 80.
Small pox, Gregory on, 166.
St. Louis university, 282.
Stomach, Prout on, 250.
Stricture of the uterus, tobacco in, 272.
Strychnia, poisonous effects of, 313.
Sulphate of quinine, toxicological effects of, 177.
Sulphur water on secretory organs, 276.
Surgery, veterinary, 353.
Sutton on inversion of the uterus, 1.

Sutton on strychnia, 313.
Syphilis, American origin of, 360.
Scarlatina, belladonna in, 75.

T

Teeth, Goddard on, 410.
Teeth, irregularities of, 72.
Tennessee, diseases of, 105.
Therapeutics, elements of, 46.
Thoracic and abdominal viscera, position of, 80.
Tic douloureux, 532.
Tobacco in stricture of the uterus, 272.
Tooth-ache, 532.
Transylvania medical school, 460.
Tubercles, seat of, 75.
Tuberculous disease, case of, 382.
Tubercles and phthisis, 455.
Typhus, ganglionic, 386.
Testis, loss of, 384.

U

University of St. Louis, 282.
Urethra, stricture of, 384.
Uterus, inversion of, 1.
Uterus, stricture of, 272.

V

Vagina, noisy expulsion of gas from, 78.
Veins, milk injected into, 177.
Veterinary surgery, 353.
Viscera, relative position of, 80.
Vital statistics of Sheffield, 171.
Voice, on the physiology of, 431.

W

Watson's lectures on the practice of physic, 228.
Williams' principles of medicine, 144.
Western medical schools, 92.
Western Journal, 188, 82.

Y

Yandell's case of stricture of the urethra, 384.
Yandell's case of epilepsy, 384.
Yandell's cases of dyspepsia, 219.
Zinc, chloride, in tooth-ache, 532.

### RECEIPTS OF THE MEDICAL JOURNAL FOR MAY.

| | |
|---|---|
| B. Razan, Greenbush, Ill., - - - - - - | $5 00 |
| D. W. Christian, Trenton, Ky., - - - - - | 5 00 |
| J. Edwards, Leavenworth, Ia., - - - - - | 5 00 |
| W. & R. T. Thornton, Calhoun, Mo. - - - - | 5 00 |
| J. Morrison, Arcadia, Ill., - - - - - - | 5 00 |
| J. B. Nash, Dixon, Ill., - - - - - - | 5 00 |
| J. G. McClain, Carthage, Tenn., - - - - | 5 00 |
| Martin & Hamilton, Clinton, Miss. - - - - | 5 00 |
| R. B. Harper, Portersville, Tenn., - - - - | 5 00 |
| C. T. Pomeroy, Buckeye, Ohio, - - - - | 5 00 |
| S. H. Dickson, Charleston, S. C., - - - - | 5 00 |
| Dr. T. Lilly, Fairfield, Ky., - - - - - | 5 00 |
| J. W. Crawford, Scotville, Ala., - - - - | 5 00 |
| S. B. Robinson, Murfreesboro, Tenn., - - - | 5 00 |
| Dr. D. Patton, Finley, Ohio, - - - - | 5 00 |
| J. J. Heady, Taylorsville, Ky., - - - - - | 5 00 |

### TO OUR SUBSCRIBERS.

We must again remind our subscribers of their arrearages to us, and *urge* them to remit their respective dues. Our expenses for the last and present volume have been more than usually heavy, whilst the receipts, we are sorry to say, have by no means augmented in like proportion. Those who owe for this Journal from the commencement, will not, we trust, suffer us to ask them again for the amounts due us. We hope, moreover, that *all* our subscribers, whether old or recent, will feel it incumbent to remit immediately.

Thus far, we have not collected enough to defray the cost of the Journal, but we are determined to sustain it. We design commencing the ninth volume, with new and improved materials—will not the subscribers encourage and sustain us by remitting forthwith by mail, at our risk, franked by the Postmaster?

*October*, 1843. PRENTICE & WEISSINGER.

The Western Journal of Medicine and Surgery is published monthly by the undersigned, at the corner of Main and Fifth streets, Louisville, at $5 per annum, payable in advance. Each number contains 96 pages, making two volumes in the year of more than 550 pages.

Contributions to its pages by the Physicians of the Valley of the Mississippi, addressed to the Editors, are respectfully solicited.

Letters on business, to be addressed, postage paid, to the publishers.

☞Postmasters, by the regulations of the Postoffice Department, will frank letters containing subscription money, and all remittances so franked are at the risk of the publishers.

*January*, 1844. PRENTICE & WEISSINGER.

